# Reconfiguration of Transcriptional Control of Lysine Biosynthesis in *Candida albicans* Involves a Central Role for the Gcn4 Transcriptional Activator

**DOI:** 10.1128/mSphere.00016-15

**Published:** 2016-01-22

**Authors:** Yumnam Priyadarshini, Krishnamurthy Natarajan

**Affiliations:** Laboratory of Eukaryotic Gene Regulation, School of Life Sciences, Jawaharlal Nehru University, New Delhi, India; Carnegie Mellon University

**Keywords:** *Candida albicans*, lysine biosynthesis, transcriptional regulation, transcriptional activator, Gcn4, Lys14

## Abstract

Evolution of transcriptional control is essential for organisms to cope with diversification into a spectrum of environments, including environments with limited nutrients. Lysine biosynthesis in fungi occurs in eight enzymatic steps. In *Saccharomyces cerevisiae*, amino acid starvation elicits the induction of *LYS* gene expression, mediated by the master regulator Gcn4 and the pathway-specific transcriptional regulator Lys14. Here, we have shown that the activation of *LYS* gene expression in the human fungal pathogen *Candida albicans* is predominantly controlled by Gcn4 under amino acid starvation conditions. Multiple lines of study showed that the four *C. albicans LYS14*-like genes have no role in the regulation of lysine biosynthesis. Whereas Gcn4 is dispensable for the growth of *S. cerevisiae* under lysine deprivation conditions, it is an essential regulator required for the growth of *C. albicans* under these conditions, as *gcn4* deletion caused lysine auxotrophy. Gcn4 is required for the induction of increased *LYS2* and *LYS9* mRNA but not for the induction of increased *LYS4* mRNA. Under lysine or isoleucine-valine deprivation conditions, Gcn4 recruitment to *LYS2* and *LYS9* promoters was induced in *C. albicans*. Indeed, in contrast to the *S. cerevisiae LYS* gene promoters, all *LYS* gene promoters in *C. albicans* harbored a Gcn4 binding site but not all harbored the *S. cerevisiae* Lys14 binding site, indicating the evolutionary divergence of cis-regulatory motifs. Thus, the transcriptional rewiring of the lysine biosynthetic pathway in *C. albicans* involves not only neofunctionalization of the four *LYS14*-like genes but the attendant strengthening of control by Gcn4, indicating a coordinated response with a much broader scope for control of amino acid biosynthesis in this human pathogen.

**IMPORTANCE** Microbes evolve rapidly so as to reconfigure their gene expression to adapt to the metabolic demands in diverse environmental niches. Here, we explored how conditions of nutrient deprivation regulate lysine biosynthesis in the human fungal pathogen *Candida albicans*. We show that although both *Saccharomyces cerevisiae* and *C. albicans* respond to lysine deprivation by transcriptional upregulation of lysine biosynthesis, the regulatory factors required for this control have been reconfigured in these species. We found that Gcn4 is an essential and direct transcriptional regulator of the expression of lysine biosynthetic genes under lysine starvation conditions in *C. albicans*. Our results therefore suggest that the regulation of the lysine biosynthetic pathway in *Candida* clade genomes involves gain of function by the master transcriptional regulator Gcn4, coincident with the neofunctionalization of the *S. cerevisiae* pathway-specific regulator Lys14.

## Abstract (precis)

Microbes evolve rapidly so as to reconfigure their gene expression to adapt to the metabolic demands in diverse environmental niches. Here, we explored how conditions of nutrient deprivation regulate lysine biosynthesis in the human fungal pathogen *Candida albicans*. We show that although both *Saccharomyces cerevisiae* and *C. albicans* respond to lysine deprivation by transcriptional upregulation of lysine biosynthesis, the regulatory factors required for this control have been reconfigured in these species. We found that Gcn4 is an essential and direct transcriptional regulator of the expression of lysine biosynthetic genes under lysine starvation conditions in *C. albicans*. Our results therefore suggest that the regulation of the lysine biosynthetic pathway in *Candida* clade genomes involves gain of function by the master transcriptional regulator Gcn4, coincident with the neofunctionalization of the *S. cerevisiae* pathway-specific regulator Lys14.

## INTRODUCTION

How genome evolution contributes to the plasticity of gene regulation even in closely related genomes is critical for the survival of microbes in diverse environments. *Saccharomyces cerevisiae* has been the model for our understanding of microbial metabolism and its regulation. Several recent studies, however, highlighted the rewiring of transcriptional regulatory circuits in *Candida albicans*, a human fungal pathogen, and other fungal genomes (for reviews, see references 1 to 3). Such rewiring of the regulatory circuits in *C. albicans* included the replacement of the Tbf1 transcription factor, as well as the cognate promoter binding sites, in the ribosomal protein regulon (4, 5), the regulation of the sexual cycle (6), the recent evolution of new binding motifs for Mcm1 (7), and the reconfiguration of the Gal4 binding site to be the Cph2 binding site in the *GAL10* gene promoter (8).

**FIG 1:**
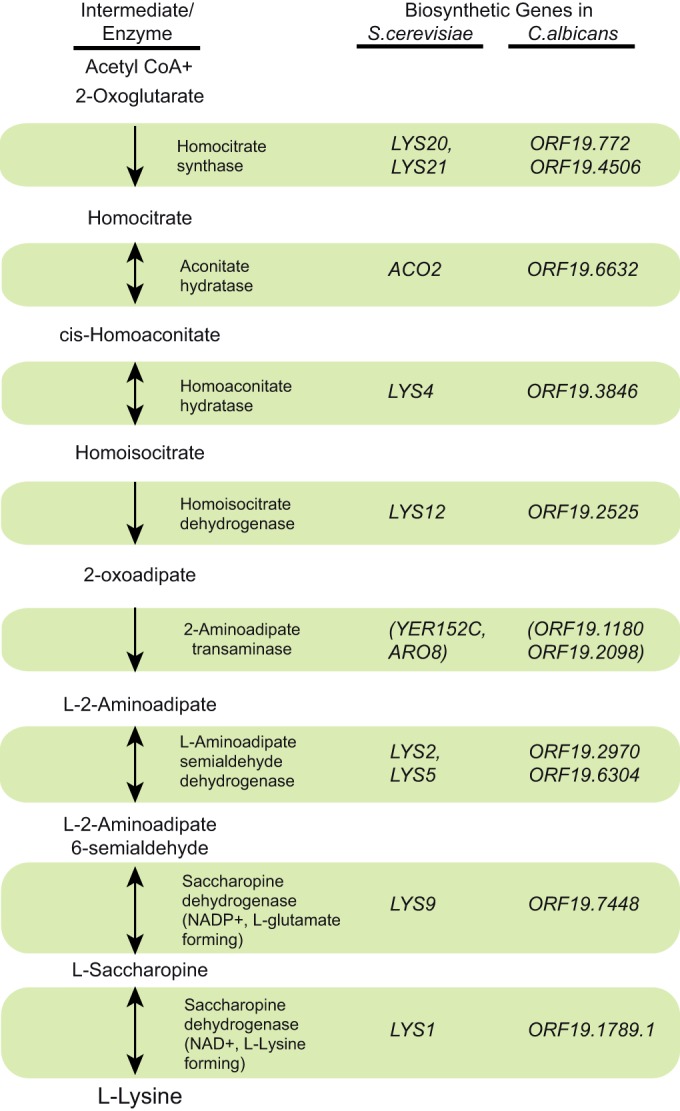
Comparison of lysine biosynthetic pathways in *S. cerevisiae* and *C. albicans*. The lysine biosynthetic pathway has 8 steps, involving 7 enzymes encoded by 12 unlinked genes. Regulation of the lysine biosynthetic pathway in *S. cerevisiae* is mediated by general amino acid control via Gcn4, feedback inhibition of homocitrate synthase activity by lysine, and induction of the lysine pathway-specific transcriptional regulator Lys14p by alpha-aminoadipate semialdehyde (15) *C. albicans LYS* genes as annotated in the *C. albicans* genome sequence are shown.

Lysine is an essential amino acid for animals and is obtained from proteins in the diet. In lower eukaryotes, including fungi, lysine biosynthesis, outlined in Fig. 1, begins with condensation of α-ketoglutarate (2-oxoglutarate) and acetyl-coenzyme A (CoA) and has seven more enzymatic steps (9–11). The genes encoding all but the 2-aminoadipate transaminase step have been identified in *S. cerevisiae* (Fig. 1) (11–14). The genes encoding 2-aminoadipate transaminase listed in Fig. 1 have been tentatively assigned as *YER152c* and *ARO8*, based on the Saccharomyces Genome Database (http://www.yeastgenome.org) and the KEGG database (http://www.genome.jp/kegg/), respectively.

The regulation of lysine biosynthesis in *S. cerevisiae* occurs at the level of both gene expression and enzyme activity (15). Whereas the first enzyme in the pathway, homocitrate synthase (Lys20 and Lys21), is regulated by lysine feedback in *S. cerevisiae*, this may not be a prevalent mechanism in all fungi, since in *Aspergillus fumigatus*, the homocitrate synthase is insensitive to lysine inhibition (16). The sixth enzyme in the pathway, l-aminoadipate semialdehyde dehydrogenase (Lys2), is activated by Lys5-mediated phosphopantetheinylation (17). The regulation of *LYS* pathway gene expression is achieved both by a pathway-specific regulator and by the general amino acid control mediated by Gcn4 (15). In *S. cerevisiae*, amino acid starvation and multiple other stress conditions lead to translational derepression of the expression of the bZIP protein Gcn4, a master regulator of the transcription of almost 73 genes belonging to every amino acid biosynthetic pathway (reviewed in reference 18). Lys14, a Gal4-like Zn(II)_2_Cys_6_ binuclear cluster transcription factor, is the pathway-specific transcriptional regulator, which is activated by the pathway intermediate 2-aminoadipate semialdehyde (19, 20). Genome-wide expression data (see supplemental Figure S1 at http://www.jnu.ac.in/Faculty/natarajan/msphere_suppl_info.pdf) showed that almost all of the lysine biosynthetic pathway genes are induced upon histidine starvation (21), as well as by isoleucine-valine starvation (22). However, the direct regulation by Gcn4 and Lys14 at the *LYS* pathway promoters is only partially understood. *MKS1* (*LYS80*), a negative regulator of the retrograde mitochondrion-to-nucleus signaling (23), was shown to downregulate lysine biosynthesis by restricting the availability of 2-oxoglutarate (24, 25).

In *Candida albicans*, the major human fungal pathogen, cloning, the expression of recombinant proteins, and enzymatic activity have been demonstrated for open reading frames (ORFs) *ORF19*.*2525* (*C. albicans LYS12* [*CaLYS12*]) (26), *ORF19*.*2970* (*CaLYS2*) (27), and *ORF19*.*6304* (*CaLYS5*) (28). Genetic studies in *C. albicans* strains bearing *ORF19.772* (*LYS21*) and *ORF19*.*4506* (*LYS22*) deletions showed a loss of homocitrate synthase activity and lysine auxotrophy (29). Heterologous complementation by the *ORF19*.*1789*.*1* (*LYS1*) gene in *S. cerevisiae* showed saccharopine dehydrogenase (NAD^+^, l-lysine-forming) activity (30) (Fig. 1). *C. albicans* genome annotation (31, 32) and comparison to *S. cerevisiae* genes led to the assignment of gene-enzyme relationships for orthologous genes encoding lysine biosynthetic enzymes in *C. albicans* (Fig. 1). However, no systematic studies have been carried out in *C. albicans* to understand the control of *LYS* pathway gene expression by amino acids, especially that by lysine deprivation.

In this study, we show that lysine deprivation elicits transcriptional upregulation of the *LYS* biosynthetic pathway in *C. albicans*. We then provide multiple lines of evidence supporting the view that the four *LYS14*-like genes, *LYS141*, *LYS142*, *LYS143*, and *LYS144*, have no role in the regulation of *LYS* gene expression in *C. albicans*. Cloning and expression of *C. albicans LYS141* (*ORF19.5548*), *LYS142* (*ORF19.4778*), *LYS143* (*ORF19.4776*), and *LYS144* (*ORF19.5380*) could not complement lysine auxotrophy of the *S. cerevisiae* lys14Δ mutant. Nor did the deletion of the four genes cause lysine auxotrophy in *C. albicans*, and none of the four were recruited to the promoters of *LYS* pathway genes under lysine deprivation conditions. Our results demonstrate that Gcn4 is essential for *C. albicans* growth under lysine deprivation conditions and, further, show that Gcn4 is a direct activator of multiple *LYS* biosynthetic pathway genes.

## RESULTS AND DISCUSSION

### *C. albicans* lysine biosynthetic pathway genes are induced by lysine starvation.

**FIG 2:**
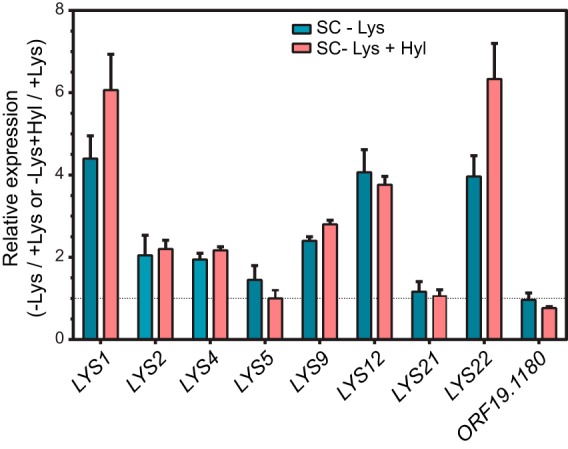
Lysine deprivation activates the expression of lysine biosynthetic pathway genes in *C. albicans*. *C. albicans* strain RPC206 was grown in SC, SC−Lys, or SC−Lys+Hyl (0.1 mM Hyl) medium, total RNA was isolated, and cDNA was prepared. qRT-PCR was carried out using SYBR green chemistry and primers specific for each of the indicated *LYS* genes. Relative expression levels, indicated as fold changes, were calculated as the ratio of the mRNA level in SC−Lys or SC−Lys+Hyl medium relative to the level in SC medium. The *C. albicans SCR1* transcript was used as an endogenous control (41) the standard errors of the means (SEM) of data from three independent biological replicates.

To determine whether the expression of *C. albicans* lysine biosynthetic pathway genes is regulated by lysine availability, we cultured cells in synthetic complete (SC) medium with lysine (SC+Lys medium) or without lysine (SC−Lys medium), as well as SC−Lys medium plus 0.1 mM 5-hydroxylysine (SC−Lys+Hyl medium), and quantified the mRNA levels of several *LYS* biosynthetic genes (Fig. 1) by quantitative PCR (qPCR) analysis. The data showed that the *LYS* genes were significantly induced upon lysine deprivation. Whereas the expression of *LYS1*, *LYS12*, and *LYS22* was highly induced (≥4-fold), the expression of *LYS2*, *LYS4*, and *LYS9* was induced ~two- to threefold. However, the expression of *LYS5*, *LYS21*, and *ORF19.1180* was unaffected by lysine deprivation (Fig. 2). It is interesting that of the two homocitrate synthase genes *LYS21* and *LYS22* in *C. albicans*, only *LYS22* was induced upon lysine deprivation. The data also showed that the expression of *LYS5*, encoding phosphopantetheinyl transferase, required for activation of the *LYS2* product, was not induced by lysine deprivation, although *LYS2* expression was itself induced (Fig. 2). In contrast, both *LYS2* and *LYS5* mRNA levels in *S. cerevisiae* were increased upon amino acid starvation (21, 22). The expression of *ORF19.1180* was not altered upon lysine deprivation (Fig. 2), and therefore, the role of this putative α-aminoadipate aminotransferase in lysine biosynthesis is unclear. In *S. cerevisiae*, Aco2 was shown to catalyze the homocitrate-to-homoaconitate conversion (11). However, a proteomic study in *C. albicans* showed that, whereas Aco1 is upregulated, Aco2 is downregulated in a Gcn4-dependent manner upon amino acid starvation (33). Thus, there appear to be differences between *S. cerevisiae* and *C. albicans* as to which of the two aconitate hydratase genes carry out this step. Overall, our data showed that *C. albicans* responds to lysine deprivation by transcriptional upregulation of the bulk of the genes in the lysine biosynthetic pathway.

### The four *LYS14*-like genes in *C. albicans* are not functional orthologs of the *S. cerevisiae* transcriptional activator *LYS14*.

**FIG 3:**
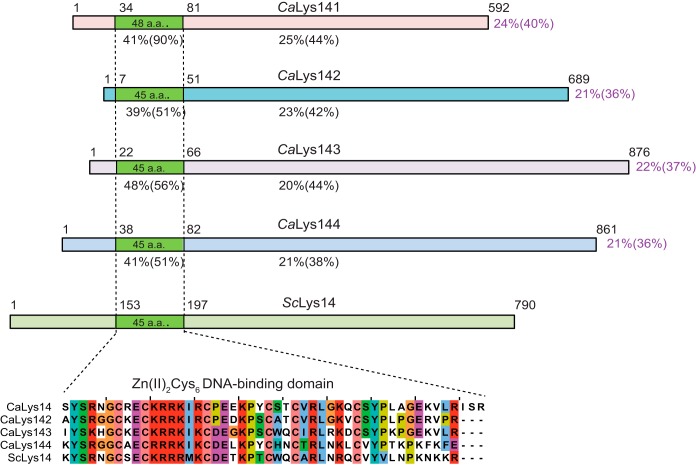
*C. albicans* Lys14-like proteins are highly related only to the Zn(II)_2_Cys_6_ DNA-binding domain of the *S. cerevisiae* transcriptional regulator Lys14. (Top) Schematic diagram showing the four *C. albicans* Lys14-like proteins, Lys141 (ORF19.5548), Lys142 (ORF19.4778), Lys143 (ORF19.4776), and Lys144 (ORF19.5380), in comparison to *S. cerevisiae* Lys14. The Gal4-like Zn(II)_2_Cys_6_ DNA-binding domain was determined using the SMART tool. The identity and similarity (in parentheses) of the Zn finger domain (green) and of the whole protein to *S. cerevisiae* Lys14 are shown for each protein. (Bottom) Multiple sequence alignment of the Zn(II)_2_Cys_6_ DNA-binding domains from *C. albicans* Lys14-like proteins and *S. cerevisiae* Lys14.

To investigate the mechanism of upregulation of the lysine biosynthetic pathway, we sought to identify the role of the *C. albicans LYS14*-like genes *LYS141*, *LYS142*, *LYS143*, and *LYS144.* Sequence analyses of the amino acid sequences encoded by the four *LYS14*-like genes showed ~40% amino acid sequence identity of the 45-amino-acid region encompassing the Zn(II)_2_Cys_6_ binuclear cluster DNA binding motif with that of *S. cerevisiae* Lys14 (ScLys14) (Fig. 3). The rest of the protein sequence had poor sequence identity (~20%) with that of ScLys14.

We employed a heterologous genetic complementation approach to identify the activity of the four *LYS14*-like genes in the regulation of the *LYS* gene pathway in *S. cerevisiae*. We cloned each of the four *LYS14*-like genes under the control of the *GAL1* promoter in the vector pGAL-HA, introduced them individually into *S. cerevisiae lys14*Δ mutant strain 3973, and tested the mutants on SC−Lys or SC+Lys plates containing glucose, raffinose, or raffinose and galactose. As expected, with reference to the growth of control strain BY4741 (wild type [WT]), the parental strain 3973 (*lys14*Δ) showed retarded growth on SC−Lys plates compared to its growth on SC+Lys plates (see supplemental Figure S2 at http://www.jnu.ac.in/Faculty/natarajan/msphere_suppl_info.pdf). Interestingly, the introduction of each of the four *LYS14*-like genes could not restore this defect on plates with SC−Lys plus raffinose and galactose (see supplemental Figure S2 at the URL mentioned above). The four *C. albicans LYS14*-like coding sequences contained multiple CUG codons (see supplemental Table S3 at the URL mentioned above) that, instead of the altered Ser in *C. albicans*, would be decoded as Leu upon expression in *S. cerevisiae*. However, none of these CUG codons was found in the zinc cluster DNA-binding domain, and their positions were also not conserved between the four *LYS14*-like coding sequences. Moreover, Western blot analyses showed that all four genes were indeed expressed in medium containing galactose but not in medium with raffinose (data not shown). These data indicated that the *C. albicans LYS14*-like genes cannot complement the lysine auxotrophy of the *S. cerevisiae lys14*Δ strain.

To investigate the role of *LYS14*-like genes in *C. albicans* under lysine deprivation conditions, we constructed homozygous deletion mutants with deletions of each of the four *LYS14*-like genes. We employed pHAH1 (34) to delete both alleles, marked with *HIS1* or *ARG4*, in the *C. albicans* parental strain SN152. The correct integration of the deletion cassettes, as well as the absence of a wild-type copy of the candidate gene, was confirmed by PCR. The *lys14-*like deletion mutant strains and a control prototrophic strain, RPC206, an isogenic derivative of SN152, were grown in SC+Lys medium and spotted onto plates with SC−Lys medium, SC−Lys+Hyl medium, or the control SC+Lys+Hyl medium, and growth was monitored at 30°C. The results showed that none of the four *lys14-*like mutant strains was impaired for growth in lysine-deficient medium (see supplemental Figure S3 at http://www.jnu.ac.in/Faculty/natarajan/msphere_suppl_info.pdf). The control *C. albicans lys2*Δ mutant strain, as expected, did not grow (see supplemental Figure S2 at the URL mentioned above). Although none of the single homozygous *lys14*-like genes is required for growth of *C. albicans* under lysine-deficient conditions *in vitro*, it was unknown whether any combination of the four *lys14*-like gene deletion mutations would confer a growth defect under lysine-deficient conditions.

**FIG 4:**
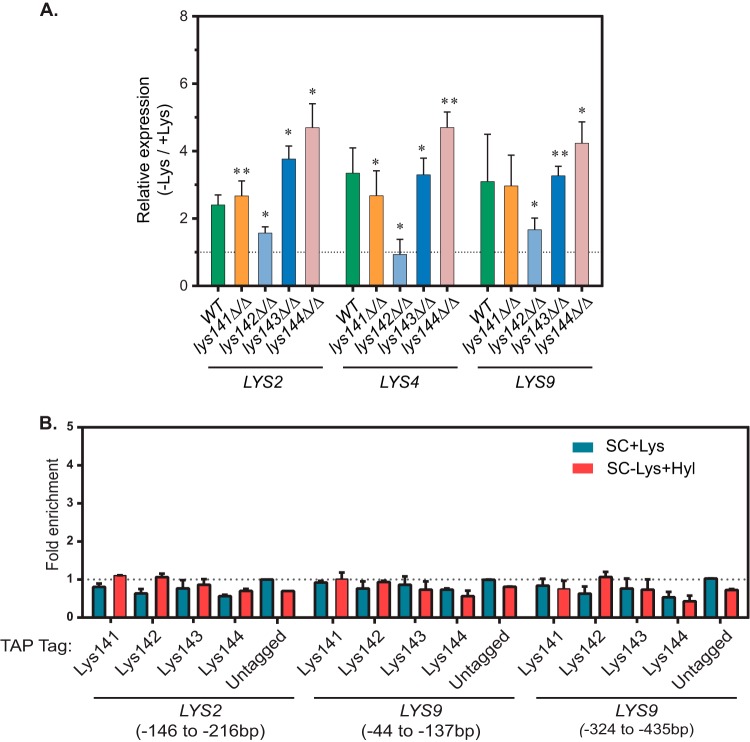
None of the four *C. albicans LYS14*-like genes are required for activation of lysine biosynthetic pathway gene expression. (A) *C. albicans lys141*Δ/Δ, *lys142*Δ/Δ, *lys143*Δ/Δ, and *lys144*Δ/Δ mutants and the control strain RPC206 were cultured in lysine-deficient (SC−Lys) or control (SC+Lys) medium, total RNA was isolated, and cDNA was prepared. Real-time qRT-PCR was carried out using primers specific for *LYS2*, *LYS4*, and *LYS9* genes, and relative expression (fold change in expression in SC−Lys medium with respect to that in SC+Lys medium) was plotted. Error bars represent the means ± SEM; *P* values (*, ≤0.05; **, ≤0.01) were calculated using Student’s *t* test in GraphPad Prism. (B) None of the four *C. albicans* Lys14-like proteins is recruited to the *LYS2* and *LYS9* gene promoters. TAP-tagged *C. albicans* strains PC205 (*LYS141*::TAP), PC206 (*LYS142*::TAP), PC209 (*LYS143*::TAP), and PC212 (*LYS144*::TAP) and the control untagged SN152 strain were grown in SC+Lys or SC−Lys+Hyl (0.1 mM Hyl) medium and cross-linked with formaldehyde, and the sonicated chromatin extracts were immunoprecipitated using IgG-Sepharose 4B. The de-cross-linked and purified DNA was analyzed by qPCR with primers specific for *LYS2* (ONC620/ONC621) or *LYS9* (ONC624/ONC625 and ONC628/ONC629) promoters or with primers ONC305/ONC306 for a noncoding region in chromosome I as a control (41) The fold enrichment of each specific target was calculated by the 2^−ΔΔ*CT*^ method from the results of at least three independent biological replicates. Error bars represent the means ± SEM.

To further probe the role of the four *LYS14*-like genes in the regulation of lysine biosynthesis, we examined whether the expression of the four *LYS14*-like genes is regulated by lysine availability. The wild-type strain RPC206 was grown in lysine-replete (SC+Lys), lysine-deficient (SC−Lys), or lysine-starved (SC−Lys+Hyl) medium, total RNA was extracted, and *LYS141*, *LYS142*, *LYS143*, and *LYS144* mRNA levels were quantified using quantitative reverse transcription-PCR (qRT-PCR). The data showed that none of the four *LYS14* mRNAs was upregulated upon lysine deprivation (see supplemental Figure S4 at http://www.jnu.ac.in/Faculty/natarajan/msphere_suppl_info.pdf). Next, we examined whether the activation of *LYS2*, *LYS4*, and *LYS9* mRNA upon lysine deprivation was dependent on any of the four *LYS14*-like genes. The qRT-PCR data showed that the *LYS2*, *LYS4*, and *LYS9* mRNAs were induced at or close to wild-type levels in the *lys141*Δ mutant (Fig. 4A). In the *lys142*Δ mutant, however, lysine deprivation led to a partial reduction of *LYS2* and *LYS9* mRNA induction and abrogation of *LYS4* mRNA induction. In the *lys143*Δ and *lys144*Δ mutants, the induction of higher *LYS2*, *LYS4*, and *LYS9* mRNA levels was greater than that in the wild type (Fig. 4A), indicating that *LYS143* and *LYS144* had a repressive effect on the three *LYS* biosynthetic genes examined here. Together, our results showed that although none of the four *LYS14*-like genes were obligate for *LYS* biosynthetic gene expression, they had differential effects on the expression of the three key *LYS* biosynthetic genes.

Next, we wished to assess whether there was a direct role for *LYS141*, *LYS142*, *LYS143*, or *LYS144* in the regulation of *LYS* biosynthetic genes under lysine deprivation conditions. We constructed *C. albicans* strains expressing TAP epitope-tagged *LYS141*, *LYS142*, *LYS143*, and *LYS144* from genomic loci. Western blot analyses showed that Lys141-TAP, Lys142-TAP, Lys143-TAP, and Lys144-TAP were all expressed at levels very comparable to each other in *C. albicans* (see supplemental Figure S5 at http://www.jnu.ac.in/Faculty/natarajan/msphere_suppl_info.pdf). Moreover, the data also showed that lysine deprivation did not alter the levels of any of the four Lys14-like proteins (see supplemental Figure S5 at the URL mentioned above), consistent with our mRNA analyses.

The TAP-tagged strains were used in chromatin immunoprecipitation assays to determine the promoter occupancy under lysine-replete and -deficient conditions. The strains were cultured, cross-linked with formaldehyde, chromatin sheared, and immunoprecipitated with IgG-Sepharose 4B to immunoprecipitate TAP-tagged proteins. The chromatin immunoprecipitation (ChIP)-qPCR data showed that none of the four regulators was bound to *LYS2* or the *LYS9* promoter regions above background levels under either lysine-replete or lysine-deficient conditions (Fig. 4B). These data are consistent with our finding that the *C. albicans LYS2* and *LYS9* gene promoters do not harbor the *S. cerevisiae* Lys14-binding sites (see supplemental Figures S6 and S7 at http://www.jnu.ac.in/Faculty/natarajan/msphere_suppl_info.pdf). Thus, our data from multiple lines of investigation suggest that the four *C. albicans LYS14*-like genes do not play any role in the regulation of the lysine biosynthetic pathway even upon lysine deprivation.

Other studies have also indicated that the *LYS14* regulators may have roles outside lysine biosynthesis regulation. We examined a set of publicly available microarray data (35) and found that the expression of *C. albicans LYS142* is upregulated during oxidative stress (data not shown). Another study employed green fluorescent protein (GFP)-tagged Lys141 and Lys144, strongly overexpressed from the *TDH3* promoter in yeast extract-peptone-dextrose (YPD) medium, and identified the genome-wide occupancy of the two regulators (36). Their data revealed that the two regulators bound to a small number of promoters, none of which were from the *LYS* biosynthetic gene pathway (36). Besides, Lys141 and Lys144 seem to make distinct contributions to *C. albicans* pathogenesis and commensalism (36). Moreover, Pérez et al. also showed that the four Lys14-like recombinant proteins have different DNA binding specificities as well (37). Thus, the studies described above, together with our results from studies carried out under lysine deprivation conditions, establish neofunctionalization of the four *LYS14*-like regulators in *C. albicans*.

### *GCN4* is an essential regulator of the lysine biosynthetic pathway in *C. albicans.*

**FIG 5:**
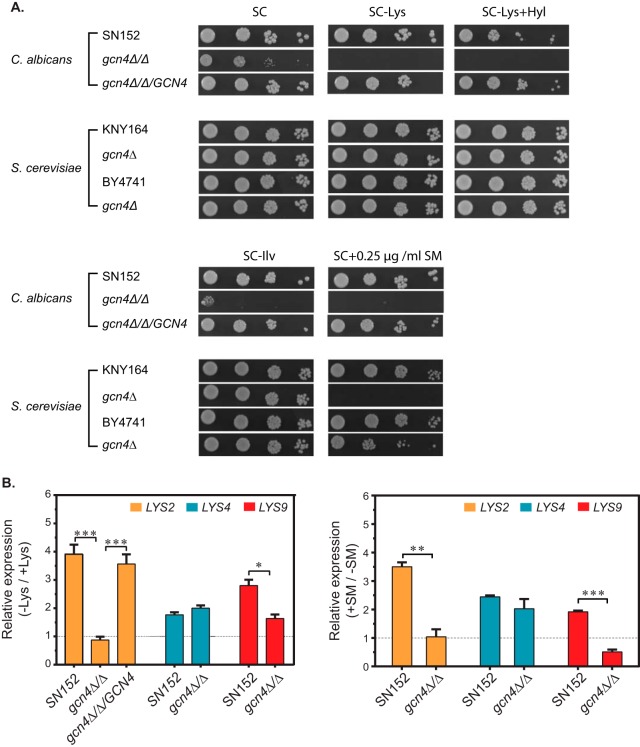
Gcn4 is an essential regulator of lysine biosynthesis in *C. albicans*. (A) *C. albicans gcn4*Δ/Δ mutant shows lysine auxotrophy. *C. albicans* strains SN152 (WT), PC234 (*gcn4*Δ/Δ), and PC291 (*gcn4*Δ/Δ/*GCN4*) and *S. cerevisiae* strains KNY164 (WT), KNY124 (*gcn4*Δ), BY4741 (WT), and 249 (*gcn4*Δ) were grown overnight in SC medium, and 10-fold serial dilutions were spotted on SC, SC−Lys, and SC−Lys+Hyl (0.1 mM Hyl) plates or on SC−Ile−Val plates containing 0.25 µg/ml or no sulfometuron methyl (SM). The plates were incubated at 30°C, and images were acquired. (B) *GCN4* is required for the activation of lysine biosynthetic gene transcription. *C. albicans* SN152, PC234 (*gcn4*Δ/Δ), and PC291 (*gcn4*Δ/Δ/*GCN4*) strains were grown either under lysine deprivation conditions (SC−Lys medium), or Ile–Val starvation conditions (SC−Ile−Val medium with 0.5 µg/ml SM) as described in Materials and Methods. Total RNA was isolated, cDNA was prepared, and transcript levels were determined by qRT-PCR. The expression levels of the *LYS* genes relative to the *SCR1* transcripts (endogenous control) under lysine deprivation conditions (left) and Ile-Val starvation conditions (right) were determined from three independent biological replicates and plotted. *P* values (*, ≤0.05; **, ≤0.01; ***, ≤0.001) were calculated using Student’s *t* test.

Past studies have shown that lysine biosynthesis in *S. cerevisiae* is regulated not only by Lys14 but also by the master regulator Gcn4 under amino acid starvation conditions (15, 21). To determine the contribution of *GCN4* in the activation of the lysine biosynthetic pathway in *C. albicans*, we first constructed a *gcn4*Δ/Δ null mutant strain. A plate growth assay showed that the *gcn4*Δ/Δ mutant, as expected from studies in *S. cerevisiae* (Fig. 5A), was impaired for growth in medium containing sulfometuron methyl, an inhibitor of the *ILV2* gene product (Fig. 5A). Remarkably, the *C. albicans* gcn4Δ/Δ mutant but not the *S. cerevisiae gcn4*Δ/Δ mutant displayed auxotrophy in lysine-deficient medium that was corrected by lysine supplementation (Fig. 5A). The integration of a cloned *C. albicans GCN4* at the native locus completely rescued the growth defect of the *gcn4*Δ/Δ mutant in medium deficient in Lys or Ile-Val (Fig. 5A). The control transformant bearing the CIp10-*LEU2* empty vector, however, failed to rescue the lysine auxotrophy (data not shown). Thus, *GCN4* is an essential regulator of lysine biosynthesis in *C. albicans*.

To assess whether lysine biosynthetic pathway gene expression is dependent on Gcn4, we carried out qRT-PCR analysis to quantify the mRNA levels of *LYS4*, *LYS2*, and *LYS9* in WT and *gcn4*Δ/Δ strains*.* We found statistically significant reductions in the upregulation of *LYS2* and *LYS9* expression in the *gcn4*Δ/Δ strain compared to their expression in the WT control both under Lys starvation and Ile-Val starvation conditions (Fig. 5B). The transcriptional activation of *LYS4*, however, was not impaired by the *gcn4*Δ/Δ mutation. Reintroduction of a cloned copy of *GCN4* into the *gcn4*Δ/Δ strain also restored the induction of *LYS2* mRNA (Fig. 5B). These data showed that Gcn4 is required for induction of the expression of at least two of the key *LYS* genes. A requirement of Gcn4 for the induction of *LYS1* (and *LYS2*) mRNA under histidine starvation conditions was shown previously, and thus, additional *LYS* genes are under Gcn4 control (38). Interestingly, our data showing that *LYS4* activation is independent of Gcn4 suggest that an additional regulator(s) responds to lysine deprivation to activate its transcription.

**FIG 6:**
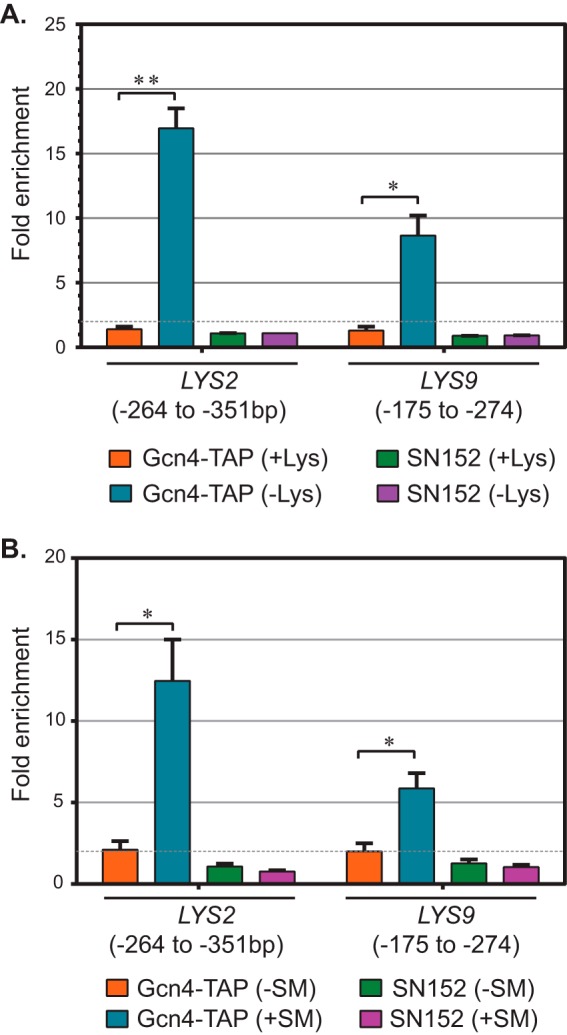
Gcn4 is recruited to *LYS2* and *LYS9* promoters under amino acid starvation conditions. Chromatin immunoprecipitation assays were carried out from chromatin extracts of *C. albicans* strains PC227 (*GCN4*::TAP) and SN152 (untagged) cultured under lysine deprivation (A) and Ile-Val starvation (B) conditions. Real-time qPCR was carried out using primers that amplified the *LYS2* (ONC622/ONC623) or the *LYS9* (ONC626/ONC627) promoter region. Fold enrichment with reference to a nonspecific region was calculated as described previously (41) the data were obtained from at least three independent biological replicates. Error bars represent SEM; *P* values of ≤0.05 (*) and ≤0.01 (**) are indicated.

To examine the mechanism of *LYS* biosynthetic gene regulation, we assessed whether Gcn4 is indeed recruited to *LYS* gene promoters upon amino acid starvation. We constructed a homozygous TAP-tagged *GCN4* strain and carried out chromatin immunoprecipitation-qPCR assays. Our data showed that Lys deprivation, as well as Ile-Val starvation, led to dramatic, statistically significant stimulation of Gcn4::TAP occupancy at both the *LYS2* and the *LYS9* promoter *in vivo* (Fig. 6A and B). A ChIP assay carried out using the untagged control strain SN152 did not yield any enrichment, indicating the specificity of the assay. Together, these results established that Gcn4 is an activator of the *LYS* biosynthetic pathway in *C. albicans*.

In summary, we have shown that lysine biosynthesis is induced by Gcn4 upon Lys and other amino acid deprivation in *C. albicans*. Using a wide variety of assays, we have demonstrated that the four paralogous *LYS14* gene family members do not directly regulate Lys biosynthesis in *C. albicans*. Importantly, we have shown that *GCN4* is an essential activator of Lys biosynthesis in *C. albicans*. Because the four *CaLYS14*-like genes do not complement the Lys auxotrophy of the *S. cerevisiae lys14*Δ mutant, we hypothesize that the DNA binding regions of the four CaLys14 sequences have diverged far enough that they no longer recognize the *S. cerevisiae* Lys14 binding sites. Consistent with this view, recent work has demonstrated that, indeed, the four *C. albicans* Lys14 regulators bind to altered DNA sequences identified by genome-wide chromatin immunoprecipitation with microarray technology (ChIP-chip), as well as by electrophoretic mobility shift assays (EMSAs) (36, 37). Moreover, the *C. albicans LYS* biosynthetic gene promoters do not harbor either the *S. cerevisiae* Lys14 binding sites (see supplemental Figures S6 and S7 at http://www.jnu.ac.in/Faculty/natarajan/msphere_suppl_info.pdf) or the *C. albicans* Lys14-binding sites.

The single Lys14 regulator found in *Saccharomyces* clade genomes has expanded in the *Candida* clade genomes to about two or three in the haploid *Candida* genomes and four or five in the diploid species, such as *C. albicans* (see Candida_Lys14_Orthologs at http://www.broadinstitute.org/cgi-bin/regev/orthogroups/show_orthogroup.cgi?orf=orf19.5548 [24 September 2015, posting date]). Coincident with the expansion of this regulatory protein family, we found that the *LYS* gene promoters in *Candida* clade genomes lacked the ScLys14 binding sites (see supplemental Figure S7 at http://www.jnu.ac.in/Faculty/natarajan/msphere_suppl_info.pdf) but retained the binding site(s) for the master regulator Gcn4, indicating that Gcn4 is evolutionarily ubiquitous and a central hub for the transcriptional control of *LYS* gene transcription. Moreover, *C. albicans* genomic annotation lacked a sequence ortholog of *MKS1* (*LYS80*), a negative regulator of Lys biosynthesis.

**FIG 7:**
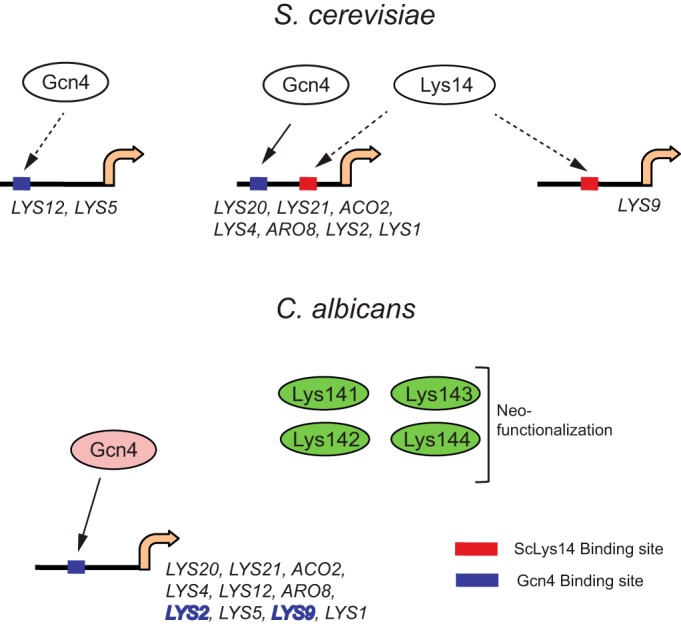
Transcriptional reconfiguration of lysine biosynthetic pathway in *C. albicans*. Schematic diagram illustrating the occurrence of binding site(s) for *S. cerevisiae* Lys14 (blue) or Gcn4 (red) in the promoters (up to −500 bp upstream from the ATG codon) of *S. cerevisiae* and *C. albicans LYS* genes, as determined using the YeTFaSCo database. The dashed arrows indicate the absence of chromatin occupancy data for Lys14 at the *S. cerevisiae LYS* gene promoters. Gcn4 occupancy was found at the> *S. cerevisiae LYS20*, *LYS2*, and *LYS1* promoters in a genome-wide ChIP-chip study (47) and at the promoters of *C. albicans LYS2* and *LYS9* in this study, as shown by the data in Fig. 6 *C. albicans* Lys14-like proteins could rescue the lysine auxotrophy in *S. cerevisiae*; besides this, no recruitment was detectable at the *LYS* gene promoters in *C. albicans*.

We examined the *S. cerevisiae LYS* gene promoters (up to −500 bp from the ATG codon) and found that 7 of the 10 *LYS* genes contain both ScLys14- and Gcn4-binding sites, whereas *LYS12* and *LYS5* contain only the Gcn4 site and the *LYS9* promoter contains only the ScLys14 binding site (Fig. 7; see also supplemental Figures S6 and S7 at http://www.jnu.ac.in/Faculty/natarajan/msphere_suppl_info.pdf). In striking contrast, all 10 of the *LYS* genes in *C. albicans* contain only the Gcn4 site (Fig. 7). Indeed, our ChIP analysis showed the recruitment of Gcn4 but not Lys14 to *LYS2* and *LYS9* promoters in *C. albicans*. Together, our results suggest that the transcriptional reconfiguration of the Lys biosynthetic pathway in *C. albicans*, and likely other *Candida* clade genomes, has been brought about by multiple evolutionary events, including alteration of regulator-binding sites in the *LYS* gene promoters, expansion and neofunctionalization of the *LYS14* regulator, and strengthening of the regulatory control by the master regulator Gcn4.

## MATERIALS AND METHODS

### Media and growth conditions.

*C. albicans* and *S. cerevisiae* strains were cultured in synthetic complete (SC) medium containing 1.5 g/liter Bacto yeast nitrogen base with ammonium sulfate plus added amino acid supplements or in yeast extract-peptone-dextrose (YPD) medium. To impose Ile-Val starvation conditions, cells were cultured at 30°C in SC medium without Leu, Ile, or Val (SC−Leu−Ile−Val medium) (for *S. cerevisiae*) or in SC−Ile−Val medium (for *C. albicans*) containing sulfometuron methyl (SM; Chem Service, PA). To impose Lys starvation conditions, cells were cultured in SC medium without lysine (SC−Lys medium) alone or in SC−Lys plus 5-hydroxylysine (dl-5-hydroxylysine hydrochloride, product number H0377; Sigma) (SC−Lys+Hyl medium).

### Strains, plasmids, and oligonucleotides.

All strains, plasmids, and oligonucleotides used are listed in supplemental Tables S1 and S2 at http://www.jnu.ac.in/Faculty/natarajan/msphere_suppl_info.pdf.

### Cloning of *CaLYS14*-like genes in pGAL-HA vector.

The pGAL-HA vector was constructed by replacing the Myc epitope tag in pESC-LEU (Agilent) with a SalI-XhoI fragment encoding a triple-hemagglutinin (HA) tag (HA_3_). The coding sequence of each of the four *CaLYS14-*like ORFs was amplified by PCR from SC5314 genomic DNA using the primer pairs ONC350/ONC351 (*LYS141*), ONC352/ONC353 (*LYS142*), ONC354/ONC355 (*LYS143*), and ONC356/ONC357 (*LYS144*) and cloned between the SalI and SmaI sites in the pGAL-HA vector, and the inserts were sequenced using primers ONC364/ONC164 and ORF-specific primers ONC362 (*CaLYS141*), ONC363/ONC364 (*CaLYS142*), ONC365/ONC366/ONC367 (*CaLYS143*), and ONC368/ONC369/ONC370 (*CaLYS144*)*.* DNA sequencing data showed that each of the four *CaLYS14-*like ORFs in the pGAL-HA constructs contained the correct sequence, bearing one of the two allelic sequences reported in assembly 22. The various *C. albicans* genome sequence assemblies, *viz*., assemblies 19, 20, and 22, revealed one or more nucleotide differences between the four *LYS14*-like ORFs, as well as between two alleles of each ORF. These allelic differences in the genome sequence and those of the cloned *LYS14*-like genes are shown in supplemental Table S3 at http://www.jnu.ac.in/Faculty/natarajan/msphere_suppl_info.pdf.

### Construction of *C. albicans* lys14Δ and *gcn4*Δ strains.

The plasmid pHAH1 (34), a modified version of the single-transformation gene deletion cassette previously described (39), was used as a template to generate gene-specific disruption cassettes using a split-marker strategy (40). Primers with homology to the gene of interest were used for amplification of the cassette from the pHAH1 plasmid in two fragments, called up-split and down-split fragments, that have ~1.0-kb overlapping regions within the *ARG4* gene. The two fragments were transformed into *C. albicans* strain SN152, and Arg-positive (Arg^+^) transformants were selected. The correct integration was confirmed by PCR, and recombinants bearing second-allele replacements by the cassette were selected on SC plates without His or Arg, thereby obtaining homozygous deletion mutants with the deletion of each of the candidate *CaLYS14* genes or the *CaGCN4* gene. The deletions were confirmed by PCR using locus-specific primers, and ORF-specific primers were used as well, to rule out any additional ORF copies.

### Cloning of *C. albicans* GCN4 and construction of *CaGCN4* add-back strain.

The *GCN4* coding sequence, flanked by 760-bp and 400-bp upstream and downstream sequences, respectively, was amplified by PCR from *C. albicans* SC5314 genomic DNA using Phusion DNA polymerase and primers ONC1011 (bearing a SalI site) and ONC1012. The PCR amplicon was digested with SalI and cloned into the CIp10-*LEU2* (41, 42) vector that had been digested with ClaI, end filled with Klenow fragment polymerase, and again digested with SalI. The positive clones were verified by BglII digestion. Plasmid pYPC24 was linearized by digestion with PacI and integrated into the *GCN4* promoter region in *C. albicans* strain PC234 (*gcn4*Δ/Δ) to generate *C. albicans* strain PC291, and the integration was confirmed by PCR.

### Construction of *C. albicans* strains bearing a C-terminal TAP tag.

We used a PCR-based one-step procedure to introduce the TAP epitope tag at the 3′ end of each ORF. Amplicons were generated from plasmid Ip21 (42) as the template, using Phusion polymerase (Fermentas) and the following primer pairs: ONC565/ONC566 (*LYS141*), ONC561/ONC562 (*LYS142*), ONC563/ONC564 (*LYS143*), ONC567/ONC568 (*LYS144*), and ONC634/ONC635 (*GCN4*). The *GCN4*::TAP*-LEU2* amplicon and each of the four *LYS14*::TAP*-LEU2* amplicons were transformed into the wild-type parental strain SN152 or the heterozygous *LYS14*/*lys14*Δ mutant strain, respectively, and selected for Leu-positive (Leu^+^) transformants. Correct integrations were confirmed by PCR using primer ONC109, a *LEU2*-TAP cassette-specific primer, and one of the following internal primers: ONC362 (*LYS141*), ONC363 (*LYS142*), ONC366 (*LYS143*), ONC369 (*LYS141*), or ONC47 (*GCN4*). To introduce the TAP tag at the second *GCN4* allele, a *HIS1-*TAP cassette in Ip22 (41) was used as the template, and the amplicon was transformed into strain PC222 and selected on SD+Arg. The correct integration was verified by diagnostic PCR using primers ONC110 (*HIS1-*TAP cassette-specific primer) and ONC47. The absence of any untagged copies of the *LYS14* ORFs or the *GCN4* ORF in the strains was also verified.

### RNA extraction and qRT-PCR analysis.

*C. albicans* strain RPC206 or the various mutant strains were precultured overnight in SC medium and diluted into SC medium, SC−Lys medium, or SC−Lys−Hyl medium (0.1 mM Hyl) and grown to an optical density at 600 nm (OD_600_) of ~2.0 at 30°C. Alternately, the precultures in SC−Ile−Val were diluted into fresh SC−Ile−Val and grown to an OD_600_ of ~0.5, and then one half of each culture was treated for 2 h with 0.5 µg/ml SM and the other left untreated. Cells equivalent to ~10 OD were harvested rapidly by filtration and snap frozen in liquid nitrogen vapor. Total RNA was isolated using the hot phenol method (43) as described previously (41), and the concentration was determined using a NanoDrop spectrophotometer.

For all RT-PCR experiments, total RNA was treated with RNase-free DNase I (Invitrogen) and used for single-stranded cDNA synthesis using a high-capacity cDNA reverse transcription kit (Invitrogen). Real-time qRT-PCR was carried out in the Applied Biosystems 7500 real-time PCR system using SYBR green PCR master mix (Applied Biosystems). The comparative cycle threshold (*C_T_*) method (2^−ΔΔ*CT*^) was used to determine the relative gene expression levels (44). *SCR1* RNA, an RNA polymerase III transcript (45), was used as an endogenous control. Control reactions without reverse transcriptase were carried out for each cDNA preparation to ascertain that no amplification would occur, as judged by high *C_T_* values (>35) and gel analysis.

### Chromatin immunoprecipitation.

*C. albicans* strains PC205 (*LYS141*::TAP), PC206 (*LYS142*::TAP), PC209 (*LYS143*::TAP), and PC212 (*LYS144*::TAP) and the control untagged SN152 strain were grown in SC+Lys medium or SC−Lys+Hyl medium (0.1 mM Hyl) and cross-linked with formaldehyde, and sonicated chromatin extracts were prepared. *C. albicans* strain PC227 (*CaGCN4*::TAP) or the untagged control strain SN152 was cultured in SC+Lys medium, SC−Lys medium, or SC−Ile−Val medium with or without SM and cross-linked with formaldehyde, and sonicated chromatin extracts were prepared. Formaldehyde (1%, vol/vol) was added to the cultures and cross-linked for 20 min at 30°C, and cells were treated to induce spheroplasts in a buffer containing 1 M sorbitol, 50 mM Tris-Cl (pH 7.4), 10 mM β-mercaptoethanol, and 0.03 mg/ml zymolyase (MP Biomedicals). Spheroplasts were resuspended in 50 mM HEPES-KOH (pH 7.5), 140 mM NaCl, 1 mM EDTA, 1% Triton X-100, 0.1% sodium deoxycholate, 0.1% SDS plus protease inhibitors (1 mM phenylmethylsulfonyl fluoride [PMSF] and 1 mg/ml each of leupeptin, aprotinin, and pepstatin) and sheared by sonication using a Bioruptor (Diagenode model UCD 300) at high power for 30 cycles (30 s on and 30 s off). The average chromatin size obtained was ~250 bp. About 25 µl of preblocked IgG-Sepharose (GE Healthcare) was used for immunoprecipitation from sheared chromatin extract prepared from cells collected from ~20 OD_600_ culture, and the ChIP assay was carried out essentially as described previously (41, 46). The cross-links in the whole-cell chromatin extracts (input) and the IP eluate were reversed and DNA purified, and enrichment determined by qPCR. ChIP-qPCR data analysis (44) and calculation of the enrichment were performed as described previously (41).
